# Investigating the Spillover Mechanisms of Payment Incentives on the Outcomes for Non‐Targeted Patients

**DOI:** 10.1002/hec.4956

**Published:** 2025-03-21

**Authors:** Philip Britteon, Søren Rud Kristensen, Yiu‐Shing Lau, Ruth McDonald, Matt Sutton

**Affiliations:** ^1^ Health Organisation, Policy and Economics (HOPE) The University of Manchester Manchester UK; ^2^ Danish Centre for Health Economics University of Southern Denmark Odense Denmark; ^3^ Centre for Health Policy Institute of Global Health Innovation Imperial College London London UK; ^4^ Alliance Manchester Business School The University of Manchester Manchester UK

**Keywords:** incentive, joint production, learning, multitasking, pay‐for‐performance, spillover

## Abstract

Payment reforms in healthcare can have spillover effects on the care experienced by non‐targeted patients treated by the same provider. Few empirical studies have quantitatively investigated the mechanisms behind these effects. We formulate theory‐driven hypotheses to investigate the spillover mechanisms of a regional payment reform in the English National Health Service, using linked patient‐physician data and difference‐in‐differences methods. We show that regional payment changes were associated with an increase in mortality of 0.321 percentage points (S.E. 0.114) for non‐targeted emergency patients who were treated by physicians with no exposure to the incentives, compared to control regions. In contrast, the mortality rate for non‐targeted patients reduced by 0.008 percentage points (S.E. 0.002) for every additional targeted patient treated per quarter by their physician. These findings were consistent across a range of sensitivity analyses. The findings suggest that providers diverted resources away from non‐targeted patients but that patients benefitted from physicians learning from the incentives. We demonstrate how the formulation of theory‐driven hypotheses about spillover mechanisms can improve the understanding of how and where spillover effects may occur, contributing to research design and policymaking.

## 1 Introduction

It is common for healthcare payers, both insurers and governments, to include explicit incentives for quality improvement in the contracts of healthcare providers (Miller and Babiarz 2014; Paris et al. 2010). In the United States, for example, linking provider payments to quality of care was a clearly stated goal of the Affordable Care Act (Burwell 2015). Despite this, calls have been made to carefully consider the design of future pay‐for‐performance schemes (Cattel and Eijkenaar 2020; Frakt and Jha 2018; Zaresani and Scott 2021). In particular, there is a growing recognition that payment changes may have unintended spillover effects on patients who were not targeted by the reform (Francetic et al. 2022).

The sign and magnitude of these spillover effects depend on multiple mechanisms, determining whether targeted and non‐targeted services are complements or substitutes to the provider (Eggleston 2005; Holmstrom and Milgrom 1991; Kaarboe and Siciliani 2011). On the one hand, providers could substitute resources and effort away from non‐targeted services in order to meet new incentives. In contrast, joint factors in production could lead to improvements in non‐targeted areas of care. New incentives could also generate learning and knowledge spillovers, leading to the wider adaptation of efficient practices (Jackson 2014; Skinner and Staiger 2015).

These spillover mechanisms have important implications when designing and assessing the effectiveness of payment reforms. Theoretical evidence suggests that financial incentives should be used with caution when spillovers could adversely impact non‐targeted areas of care (Holmstrom and Milgrom 1991; Kaarboe and Siciliani 2011). Evaluations may have also misidentified the true impact of past reforms if spillover effects on non‐targeted areas were overlooked or affected the control group used in the analysis (Rubin 1990).

Despite this, evidence on the spillover effects of payment reforms in healthcare is limited. Evaluations have tended to only present spillover effects of healthcare reforms when statistically significant or to account for unexpected findings (Francetic et al. 2022). Moreover, there exists limited empirical evidence on the mechanisms through which observed spillover effects may have occurred, beyond suggestions hypothesized in the discussion (Cattel and Eijkenaar 2020; Eijkenaar et al. 2013; Kondo et al. 2015; Zaresani and Scott 2021).

We address this limitation by analyzing the spillover mechanisms of a regional payment reform in the English National Health Service (NHS), referred to as Advancing Quality. In doing so, we make three contributions to the literature.

First, the data we use allows us to link patient records to the physicians responsible for their care. This allows us to distinguish between spillover effects on non‐targeted patients treated by physicians who were directly exposed to the incentives, physicians who indirectly exposed to incentives (i.e. working with other exposed physicians in the same specialty), and physicians with no direct or indirect exposure.

Second, the granularity of our data allows us to form theory‐driven and testable hypotheses about the mechanisms through which spillovers may occur. In particular, we consider how different spillover mechanisms, such as effort diversion or knowledge spillovers, would likely present themselves and empirically test for their existence in our data using a difference‐in‐differences model. In doing so, we provide an empirical framework that unlike previous studies, is less likely to spuriously label changes in quality for non‐targeted areas as spillovers.

Finally, we examine spillover effects in a single payer setting. Previous evidence has largely focused on spillover effects in the context of multiple payers, demonstrating that payment changes implemented by one payer can affect patients whose care was paid for by another (e.g. Baicker et al. 2013; Einav et al. 2020; McWilliams et al. 2013). By analyzing spillovers a single payer setting, we are able to examine spillover effects beyond a narrowly defined patient group and rule out the possibility that observed changes were driven by the actions of other payers in the market.

Using these data and approach, we demonstrate that the payment change led to a small but statistically insignificant increase in the mortality of non‐targeted patients admitted for a range of emergency conditions. We present evidence suggesting that this change was driven in part by sorting of non‐targeted patients within hospitals but also the substitution of resources at the organization level. In contrast, we also observe a marginal reduction in mortality rate for non‐targeted patients who were treated by physicians with higher direct exposure to the incentives suggestive of a learning effect in response to the payment change. We support these findings with a range of sensitivity analyses to demonstrate that the findings are likely to have been causally related to the rollout of incentives and therefore, can be seen as spillover effects of the payment change.

## 2 Methods

### 2.1 Institutional Setting

#### 2.1.1 The English National Health Service

In the English National Health Service (NHS), care is financed through general taxation and is free for patients at the point of delivery. Under this system, healthcare funding is allocated by the Department of Health to acute hospitals trusts and other healthcare services via local commissioning bodies.^1^ Contracts between local commissioners and trusts are partially based on activity and reimbursed under the national activity‐based financing tariff system using Healthcare Resource Groups (HRGs) (Busse et al. 2011). Doctors, nurses and other healthcare staff within these trusts are all salaried employees.

Although hospitals and staff are not directly profit‐maximizing, hospitals still face strong incentives to provide care within a financial budget. These underlying financial incentives have been extensively exploited by the Department of Health to direct the performance of the NHS at a national level (e.g. Farrar et al. 2009; Street and Maynard 2007). Alongside national initiatives for quality improvement, regional organizations within the NHS have also introduced local quality improvement schemes (Kristensen et al. 2013). One of the earliest examples of these schemes, and the focus of our study, was the Advancing Quality scheme.

#### 2.1.2 The Advancing Quality Scheme

The Advancing Quality (AQ) scheme was a pay‐for‐performance scheme introduced in the North‐West region of England in October 2008 (McDonald et al. 2015). All 24 eligible hospital trusts in the region voluntarily participated in the scheme. The scheme was an English adaptation of the US Premier Hospital Quality Incentive Demonstration (HQID) scheme (Jha et al. 2012). It initially rewarded hospitals based on the quality of care provided to patients that were admitted for one of three emergency conditions (acute myocardial infarction, heart failure, pneumonia) or patients that underwent one of three elective procedures (coronary artery bypass graft, hip replacement, and knee replacement).

The scheme assessed the performance of the participating hospitals using a set of 25 process measures (Table A1). In addition to these process measures, three outcome measures were also targeted by the scheme, including the mortality rate for patients with acute myocardial infarction. The incentivized measures were adapted from the HQID scheme in the US and targeted the care provided to patients at different stages during their stay in hospital.

Throughout the first year of the scheme, hospitals received a financial bonus dependent on how they performed in relation to the other hospitals in the region. During this time, hospitals scoring in the highest or second highest quartile received a 4% or 2% bonus payment on top of the amount reimbursed to the hospital for the associated activity under the national activity‐based financing tariff. From October 2009 to March 2010, hospitals then received an attainment bonus if their achievement in the second year of the scheme was above the median level of achievement from the first year of the scheme. In April 2010, the Advancing Quality scheme was then incorporated as a set of region‐specific requirements in a national quality incentive scheme, known as the Commissioning for Quality and Innovation (CQUIN) program. Under the CQUIN program, Advancing Quality payments were no longer framed as a reward. Instead, a fixed proportion of income was withheld and only paid to hospitals that attained a certain level of achievement based on their quality score from the first year of the scheme.

Despite changes in the design of the incentive scheme, the total amount of money potentially linked to the performance of the participating hospitals remained constant at £3.2 million per year throughout the study period. These payments were moderate in magnitude in comparison to other financial incentive schemes, equating to 2%–4% of the revenue received for the incentivized activity (Meacock et al. 2014). Payments were made directly to the hospital, but it was agreed by hospital executives that bonuses would be invested in the clinical and specialty teams whose performance had earned the bonus in order to encourage frontline buy‐in.

The AQ scheme also included other mechanisms to provide support and increase participation in the scheme. Hospitals received centralized support throughout the program, face‐to‐face training, and software tools adapted from the delivery of the Hospital Quality Incentive Demonstration scheme in the US. As the program developed, AQ leads and specialist nurses were also recruited to provide local level support within each organization. The work undertaken by AQ leads involved raising awareness of the scheme, educating staff about the treatment pathways for AQ patients, and feeding back performance data to staff involved in AQ pathways. Performance data were also published online to encourage competition between the participating hospitals and physicians.

#### 2.1.3 Previous Evidence

A handful of empirical studies have investigated the impact of the AQ scheme. McDonald et al. (2015) conducted over 400 qualitative interviews and observations from April 2009 to March 2014. Their study found providers were responsive to the financial and reputational incentives generated by the scheme. In particular, the competitive design of the AQ reward structure was identified as a key driver of change, exerting considerable pressure on AQ leads and subsequently, front‐line clinicians. Constructive feedback on performance measures and public reporting were also used to engage physicians and, in extreme cases, to shame them. However, institutional changes in behavior were not immediate, requiring AQ leads to regularly monitor physicians and retrain junior doctors on short‐term placements. There was also evidence that hospitals adapted care pathways and invested in staff, coding, and audits to meet specific AQ requirements. However, investment was often used in response to poor performance rather than as a reward for high performance as initially planned.

Studies have also quantitatively analyzed the impact of the AQ scheme. The first of these studies identified a statistically significant reduction in the mortality rate for incentivized patients during the first 18 months of the scheme (−0.88% points; *p*‐value < 0.01), using a difference‐in‐differences (DiD) model (Sutton et al. 2012). This change corresponded with improvements in the process measures used to assess performance under the AQ scheme (McDonald et al. 2015). Further evidence on the short‐term impact of AQ scheme identified a smaller impact when re‐estimated using DiD with matching (−0.74% points; *p*‐value 0.02) and the synthetic control method (−0.45% points; *p*‐value 0.21) (Kreif et al. 2016). These methods allowed the effects of unobserved variables to change over time by conditioning on past outcomes, but can produce spurious estimates when the number of pre‐policy periods is small (O’Neill et al. 2016). Finally, Kristensen et al. (2014) found no evidence of a long‐term impact of the AQ scheme from April 2010, when using the same methodology as Sutton et al. (2012).

The same studies also examined the presence of spillover effects on patients with one of six non‐targeted conditions: acute renal failure; alcoholic liver disease; intracranial injury; paralytic ileus and intestinal obstruction without hernia; duodenal ulcer; and pulmonary embolism.^2^ Mortality rates worsened for these patients in the first 18 months of the scheme compared to the rest of England (0.39% points; *p*‐value 0.30) and a synthetic control group (0.90% points; *p*‐value 0.02) (Kreif et al. 2016; Sutton et al. 2012). In contrast, Kristensen et al. (2014) observed a statistically significant reduction in the mortality rate for patients with alcoholic liver disease and acute renal failure in the longer‐term. We develop these findings by estimating the spillover effects of the AQ scheme on a wider range of non‐targeted emergency conditions and investigating the potential mechanisms behind these effects.

### 2.2 Potential Spillover Mechanisms

Payment reforms may affect the production of care beyond the targeted patient population. We define these spillovers or externalities as the effect of treatment on a non‐targeted population group or task (Angelucci and Maro 2016; Francetic et al. 2022). Spillovers can occur through multiple potential mechanisms which when combined, can determine the overall effect of a reform (Baicker et al. 2013). In the following, we outline the different spillover mechanisms that have been hypothesized in the theoretical literature and discuss their potential implications in the context of the AQ scheme.

#### 2.2.1 Effort or Resource Diversion

First, the multitasking agency literature suggests that agents may respond to incentives in one dimension of performance by substituting effort or resources away from other non‐incentivized dimensions (Eggleston 2005; Holmstrom and Milgrom 1991; Kaarboe and Siciliani 2011). These substitution or diversion effects are particularly relevant in healthcare, where providers are responsible for the care of multiple patients on a day‐to‐day basis, but subject to strict time and resource constraints (Chalkley and Malcomson 1998).

It is plausible that providers may have responded to the AQ scheme by reallocating both resources and effort away from patients with a non‐targeted condition in order to increase their revenue. On the one hand, physicians may have reallocated time and effort toward targeted patients to detriment of other patients. We refer to this particular mechanism as *effort diversion*. For example, the reporting and coding of incentivized tasks was observed to have required additional inputs from physicians and may have taken time away from patient care (McDonald et al. 2015).

Alternatively, hospitals may have prioritized incentivized patients when distributing shared resources at the organization or department level. We refer to this mechanism as *resource diversion*. Unlike effort diversion, the reallocation of resources may have affected all patients with a common care pathway, regardless of the physician responsible for their treatment. For example, some hospitals reconfigured care pathways to improve the time taken to follow‐up on targeted patients (McDonald et al. 2015). These changes may have been to the detriment of other patients if equipment and treatment facilities were prioritized for incentivized tasks.

#### 2.2.2 Joint Production

In contrast, targeted and non‐targeted dimensions of performance may have shared joint factors in production (Kaarboe and Siciliani 2011; Mullen et al. 2010; Sherry et al. 2017). Joint production, also referred to as commonalities in production, exists when the outputs of multiple services are associated with a common input or cost. When this is the case, improvements in one dimension of quality can also lead to improvements in another.

This spillover mechanism depended on the availability of additional investment or the capacity of hospitals to improve care with fixed resources. For example, the recruitment of AQ leads and specialist nurses in response to the incentives may have freed up capacity elsewhere in the system. Similarly, non‐targeted patients may have benefitted from efforts to reduce inefficiencies in the system, concerning antibiotics prescriptions, patient care pathways, and the provision of discharge instructions to patients (McDonald et al. 2015). These changes may have benefitted non‐targeted patients at both the organization and department level.

#### 2.2.3 Learning and Knowledge Transfers

Health outcomes for non‐targeted patients may have also improved if physicians learned from the incentives (Chandra and Staiger 2007). For example, physicians may have learned from the incentivized tasks and adopted new practices in other areas of their work. We refer to this mechanism as “learning by doing” in line with the wider literature. Indeed, respondents to previous qualitative interviews all described processes of adaptation and experimentation in response to the AQ scheme (McDonald et al. 2015). However, changing the behavior of physicians was observed to be challenging in the short run, with AQ leads having to regularly educate and prompt members of staff.

Knowledge may also have diffused between physicians that regularly interacted within the same network (Landon et al. 2012; Skinner and Staiger 2015). For example, knowledge may have spread between physicians regularly working in the same hospital department. We refer to this mechanism as a knowledge transfer. This learning mechanism relates to the literature on peer effects (Pollack et al. 2015) and network interactions (Jackson 2014). Previous qualitative evidence identified cases of improved collaboration and communication between previously disparate groups of staff (McDonald et al. 2015). However, there was also evidence of a lack of awareness of AQ outside the teams directly involved in the scheme.

#### 2.2.4 Patient Sorting

Finally, hospitals may have reallocated patients to different physicians and specialties. We refer to this mechanism as patient sorting (Doyle et al. 2010; Li and Waibel 2021; Xu et al. 2023). The potential impact of this spillover mechanism may have been positive or negative, depending on the allocative efficiency of patients to physicians and specialties prior to the reform.

For example, qualitative evidence observed that hospitals re‐coordinated care pathways to address concerns that AQ patients were being allocated to wards based on bed availability rather than appropriateness of care (McDonald et al. 2015). This response may have also benefitted non‐targeted patients if they were subsequently matched to more appropriate physicians and specialties. In contrast, non‐targeted patients have been allocated to physicians with less experience or to different wards, if targeted patients were prioritized during the admission process.

### 2.3 Data

#### 2.3.1 Estimation Sample

We used patient‐level data from Hospital Episode Statistics (HES) linked with mortality data from the UK Office of National Statistics (ONS) (NHS Digital 2013; Office for National Statistics 2013). HES data contained clinical, personal, administrative, and geographical information on all NHS‐funded patients admitted to a hospital in England, as well as information identifying the physician and specialty responsible for the care of each patient. ONS mortality data recorded information on all registered in‐hospital and out‐of‐hospital deaths in England and Wales.

We used data on patients admitted to hospital between October 2007 and September 2011. This study period covered one year before and three years after the introduction of the AQ scheme. We limited the follow‐up period to September 2011 because similar incentives were later adopted by other regions around this time.

We focused on patients aged 19 or over and who were admitted to an NHS hospital on an emergency basis for one of 50 non‐targeted conditions (Table A2). We identified non‐targeted conditions from the Clinical Classification System (CCS) categories used to calculate the Hospital Standardised Mortality Ratio (HSMR) (Aylin et al. 2009). Deaths from these conditions contributed to around 80% of in‐hospital deaths in England. Each CCS category grouped diagnosis codes from version 10 of the International Classification of Diseases (ICD10) that related to a specific condition or group of similar conditions. We assigned patients to each CCS category based on the primary ICD10 code recorded in the initial episode of the patient's stay in hospital.

We excluded patients from the sample if they were also diagnosed with one of the three emergency conditions incentivized by the AQ scheme (acute myocardial infarction, heart disease, and pneumonia). We identified these instances using the primary and secondary ICD10 diagnosis codes recorded during the patient's stay in hospital. This excluded admissions for a non‐targeted condition that may have also been incentivized under the AQ scheme.

To avoid counting the same death more than once in the analysis, we excluded admissions that occurred after the first emergency admission within the last 30 days of a patient's life. This attributed the death of a patient to the hospital and physician that was responsible for their care in the earliest recorded stay.

Finally, we restricted the estimation sample to patients who, on admission to hospital, were treated by a physician that also treated at least one non‐targeted patient from the same hospital trust in every quarter. We identified physicians using data on the consultant team that were allocated to the patient in the first episode of their spell in hospital, as recorded in HES. Each consultant team was led by a single physician who was responsible for the care of the patient but also included other staff involved in their treatment and care. This restriction ensured that the analysis was estimated on a balanced panel of physicians within each hospital trust.

The final estimation sample included 3,687,573 (61.8%) of the 5,965,840 patients treated for one of 50 non‐targeted conditions between October 2007 and September 2011. These patients were treated by 6601 physicians across 144 hospital trusts with at least one non‐targeted patient in every quarter. The estimation sample was similar to the full population of patients in England in terms of their mortality rate, sex, age, number of coexisting comorbidities, and the income deprivation in their area of residence (Table A3). However, the characteristics of the physicians and hospitals included in the estimation sample differed slightly due to the inclusion criteria imposed in the study.

#### 2.3.2 Variables of Interest

##### 2.3.2.1 30‐day Mortality Rate

We assessed the impact of the AQ scheme on the 30‐day mortality rate for patients admitted to hospital with a non‐targeted emergency condition. The same outcome was incentivized under the AQ scheme for patients admitted for acute myocardial infarction and was consistent with the outcome used in the previous evaluations of the scheme (Kreif et al. 2016; Kristensen et al. 2014; Sutton et al. 2012). Unlike previous studies, however, we used information on both in‐ and out‐of‐hospitals deaths recorded by the UK Office of National Statistics (ONS).

##### 2.3.2.2 Physician Exposure to the Incentives

We also exploited information on the allocation of targeted patients between physicians in the sample. This allowed us to compare differences in the spillover effects of the AQ scheme between non‐targeted patients depending on the exposure to the incentives of their physician.

First, we measured the direct exposure of physicians to patients with a targeted condition ($X_{ph}$). We measured direct exposure using the average number of targeted patients treated by each physician per quarter throughout the post‐policy period. A patient was identified as targeted if they had a primary or secondary ICD10 diagnosis code relating to one of the three incentivized emergency conditions (acute myocardial infarction, heart disease, and pneumonia).

Second, we calculated the indirect exposure to the incentives of each physician ($Z_{ph})$. We measured indirect exposure as the weighted average direct exposure of other physicians working in the same hospital specialties, using the following equation:

(1) $$Z_{ph}=\sum_{s=1}^{S}\frac{n_{p}^{s}}{N_{p}}\cdot \left[\sum_{\begin{array}{c} q=1\\ q\neq p \end{array}}^{P}\frac{n_{q}^{s}}{N^{s}-n_{p}^{s}}{\cdot X}_{q}\right]$$

where, $X_{q}$ was the direct exposure to patients with a targeted condition of another physician *q* (as defined above); $n_{p}^{s}$ and $n_{q}^{s}$ were the number of patients treated in specialty s throughout the post‐policy period by physician *p* and *q* respectively; $N_{t}^{s}$ were the total number of patients treated in specialty s throughout the post‐policy period, and $N_{p}$ was the number of patients treated by physician *p* throughout the post‐policy period.

We identified the specialties in which each physician worked based on the specialties in which their patients were treated, using the divisions of clinical work defined by the Royal Colleges and Faculties and recorded in HES (NHS 2022).

##### 2.3.2.3 Covariates

We also included information on patient characteristics and the features of their admission. These variables were used to adjust for variation in case‐mix over time that may have explained differences in the mortality rate between incentivized and non‐incentivized hospital trusts. We derived patient level variables from HES using information recorded in the first episode of a patient's stay in hospital. The variables included: an interaction between age measured in 10 years bands and the sex of the patient; whether the patient was admitted to hospital from their usual place of residence; whether the patient was admitted to hospital through the accident and emergency department of the same hospital; the day of the week a patient was admitted; and 31 binary variables indicating whether the patient had a secondary diagnosis relating to each Elixhauser comorbidity (Elixhauser et al. 1998). We further linked information on the Index of Multiple Deprivation score of the patient's area of residence (McLennan et al. 2011).

We also constructed hospital level variables measuring the volume of emergency and elective admissions and the volume of admissions for each non‐targeted condition per quarter. These variables were used in the analysis to adjust for differences in the volume and composition of hospital activity over time. In addition, we linked time invariant information on the type of hospital trust and whether the hospital trust had foundation status or not. We adjusted for the time‐varying effect of these factors in the analysis.

Finally, we constructed a set of physician and specialty level covariates using HES records. These variables included: the volume of emergency and elective admissions per quarter; the volume of non‐targeted admissions for each condition per quarter; a set of indicators identifying the specialty in which the patient was treated; and a binary variable that indicated whether the patient was treated by a physician in their contracted specialty. These variables were used in the analysis to adjust for differences in the caseload, characteristics, and allocation of non‐targeted patients to different physicians and specialties over time.

### 2.4 Empirical Analysis

#### 2.4.1 Average Spillover Effect

We first estimated the average spillover effect of the AQ scheme on the mortality rate for patients with a non‐targeted condition using a difference‐in‐differences (DiD) model. We estimated the DiD model with time‐vary covariates using the two‐stage estimation procedure proposed by Caetano et al. (2022). We estimated both stages of the analysis using ordinary least squares regression with cluster‐robust hospital level standard errors.

In the first stage of the analysis, we estimated untreated potential outcomes using the following two‐way fixed effects model. This model predicted mortality for patients admitted for a non‐targeted condition using data from the rest of England only.

(2a) $$Y_{iht}=A_{h}^{\prime }\alpha_{t}+B_{ht}\beta +C_{iht}\;\gamma +\eta_{h}+\sigma_{t}+\varepsilon_{iht}\;|\;NW_{h}=0$$

where, $Y_{iht}$ was a binary variable that indicated whether non‐targeted patient *i* died within 30 days of being admitted to hospital trust *h* in quarter *t*; $A_{h}$ was a vector of time‐invariant hospital level factors, $B_{ht}$ was a vector of time‐varying hospital level factors; $C_{iht}$ was vector of patient characteristics including features of their admission; $\eta_{h}$ were hospital fixed effects; $\sigma_{t}$ were quarter fixed effects; and ${NW}_{h}$ was a binary variable that indicated whether the patient was admitted to an incentivized hospital trust in the North‐West of England or not.

We then recovered parameters from the first stage to predict the expected probability of mortality for non‐targeted patients in the North‐West of England. Using this information, we estimated changes over time in the difference between the actual and expected probability of mortality (${\tilde{Y}}_{iht}$) for non‐targeted patients admitted to an incentivized hospital trust:

(2b) $${\tilde{Y}}_{iht}=\delta D_{t}+\eta_{h}+\varepsilon_{iht}\;|\;NW_{h}=1$$

where, $D_{t}$ was a binary variable that indicated whether the patient was admitted to hospital in the post‐policy period (October 2008 to September 2011).

This two‐stage approach was preferred to directly adjusting for covariates in a single DiD model as changes in patient and hospital level characteristics in response to the AQ scheme were excluded from the adjustment step (Caetano et al. 2022). This allowed us to adjust for changes in the level of time‐varying covariates affected by the scheme and variation in the paths of untreated potential outcomes between hospitals with different time‐invariant factors.

The coefficient δ in the model estimated changes in the difference between the actual and expected probability of mortality within 30 days for non‐targeted patients in the North‐West of England, following the AQ scheme. We interpreted this coefficient as the overall average spillover effect of the AQ scheme under the conditional parallel trends assumption (Caetano et al. 2022). This assumption required that the mortality rate for incentivized hospital trusts would have followed the same trend as the predicted expected mortality rate in the absence of the scheme (i.e. the same mortality trend as non‐incentivized hospital trusts with similar characteristics and patient populations as modeled in the first stage of the analysis). In addition, the model assumed mortality trends observed in the rest of England and during the pre‐policy period were unaffected by the introduction of the AQ scheme. This may not have been the case if hospitals anticipated and responded to the AQ scheme before its introduction or if there were between‐region spillover effects of the scheme (Butts 2023; Roth et al. 2023).

#### 2.4.2 Adjusting for Patient Sorting

Second, we investigated whether hospitals responded to the AQ scheme by reallocating non‐targeted patients to different specialties and physicians within the hospital trust (i.e. patient sorting). To do so, we re‐estimated the initial DiD analysis, including specialty and physician time‐varying covariates in the first stage of the model (see data section). These variables adjusted for changes in mortality associated with the characteristics of the specialties and physicians allocated to non‐targeted patients over time. In addition, we included physician‐hospital level fixed effects in both stages of the model to adjust for changes in the allocation of patients to physicians with different unobserved time‐invariant factors (e.g. underlying experience).

We then interpreted changes in the coefficient of interest (δ) in the second stage of the analysis to deduce whether adjusting for specialty and physician level factors impacted the average spillover effect of the scheme. We expected the estimated average spillover effect of the scheme to reduce after adjusting for specialty and physician level factors if patient sorting had an adverse impact on the mortality rate for non‐targeted patients (e.g. if patients were allocated to physicians with less experience).

#### 2.4.3 Decomposing Spillover Mechanisms

Third, we estimated differences in the spillover effect of the AQ scheme between non‐targeted patients depending on whether the physician responsible for their care was directly exposed, indirectly exposed (i.e. working with other exposed physicians), or had no exposure to the incentives. This approach allowed us to distinguish between within‐physician (effort substitution vs. learning effect), between‐physician (knowledge transfer), and organization level (resource substitution vs. joint production) potential spillover mechanisms.

To do so, we first adjusted for the time‐varying effect of being treated by a physician with higher direct ($X_{ph}$) or indirect ($Z_{ph}$) exposure to patients with a targeted condition, using data from the rest of England only. We adjusted for the time‐varying effects of these variables in the first stage of the analysis, building on the previous model which adjusted for patient sorting. This step allowed untreated potential outcomes to vary between physicians with different levels of exposure, when predicting the expected probability of mortality for patients admitted to a hospital in the North‐West of England. This ensured that we compared patients who were treated by physicians with similar levels of exposure across incentivized and non‐incentivized hospital trusts.

We then estimated the extent to which changes in the difference between the actual and expected probability of mortality (${\tilde{Y}}_{iht}$) varied between non‐targeted patients depending on the exposure of their physician. We estimated these differences in the second stage of the analysis, using the following model:

(3) $${\tilde{Y}}_{ipht}=\theta D_{t}+\mu \left(X_{ph}*D_{t}\right)+\varphi \left(Z_{ph}*D_{t}\right)+\pi_{ph}+\varepsilon_{ipht}\;|\;NW_{h}=1$$

where, $\pi_{ph}$ were physician‐hospital fixed effects.

In this model, the coefficient θ estimated the average spillover effect of the AQ scheme on the mortality rate for non‐targeted patients who were treated by a physician with zero exposure to patients with a targeted condition ($X_{ph}=0,Z_{ph}=0$). In contrast, the coefficients μ and φ estimated the marginal spillover effect of being treated by a physician who was directly or indirectly exposed to one additional targeted patient per quarter throughout the post‐policy period.

We interpreted these coefficients as the spillover effects of the scheme under additional assumptions. First, the model further required that in the absence of the scheme, differences in mortality between patients treated by physicians with higher exposure to the incentives would have followed the same pattern as differences in the expected mortality rate (i.e. that the conditional parallel trends assumption held across patients treated by physicians with different levels of exposure). Second, the model assumed that differences in the impact of the AQ scheme between physicians with higher direct and indirect exposure to the incentives were linear, separable, and additive (i.e. that the marginal spillover effect of being treated by a physician with higher exposure to the incentives followed the same functional form as specified in the model).

#### 2.4.4 Heterogeneity of Spillovers

We further stratified the analysis by whether a patient was admitted for a non‐targeted condition in the same diagnosis area as a targeted condition or not. This allowed us to investigate whether spillovers were driven by condition‐specific changes in response to policy or by changes that were applicable across all of the non‐targeted conditions included in the sample. We identified whether a non‐targeted condition was from the same diagnosis area as a targeted condition using the multilevel groupings from the CCS tool (Agency for Healthcare Research n.d.). These clinical areas included diseases of the circulatory and respiratory system.

In addition, we stratified the analysis based on whether the share of targeted patients admitted to each hospital was above or below the median share in the North‐West of England. This allowed us to investigate whether spillover effects differed between hospital trusts depending on the proportion of their total revenue affected by the scheme.

#### 2.4.5 Robustness Checks

##### 2.4.5.1 Pre‐Trends Test

To test the plausibility of the conditional parallel trends assumption, we estimated, plotted, and jointly tested for differences in the average and marginal spillover effects of the AQ scheme in each quarter throughout the pre‐policy period. This allowed us to visually and quantitatively examine the plausibility of the conditional parallel trends assumption in the period before the AQ scheme was introduced (Roth et al. 2023). As an additional check, we also estimated and tested for linear differences in estimates throughout the pre‐policy period.

##### 2.4.5.2 Alternative Model Specifications

We also tested the sensitivity of the analysis to different model specifications. First, we re‐estimated the analysis including a measure of specialty exposure to the incentives ($X_{sh}$). We measured specialty exposure as the average volume of targeted patients treated in the specialty where each non‐targeted patient was treated. This specification tested whether changes in mortality for patients treated by a physician with no exposure to the incentives were driven by organization or specialty level spillover effects (i.e. whether spillover effects differed between specialties with higher levels of exposure to the incentives). This may have been the case if resources were substituted or jointly produced at the department rather than organization level.

Second, we tested for an interaction effect between a physician's direct and indirect exposure to the incentives. This test examined whether the impact of the AQ scheme differed between non‐targeted patients who treated by a physician with high direct and indirect exposure to the incentives, relaxing the assumption that marginal spillover effects were additive.

Third, we included squared terms of a physician's direct and indirect exposure to the incentives in the model to test whether marginal returns to exposure were increasing or diminishing. For example, there may have been diminishing benefits of learning from doing as the volume of targeted patients treated by a consultant team increased. This test relaxed the assumption that increases in the volume of targeted patients treated by a physician or other physicians in the same specialty were linearly associated with changes in the mortality rate.

Fourth, we used an alternative measure of indirect exposure that measured the average exposure to the incentives of other physicians working in the same specialty *and* treating the same conditions. This alternative measure identified potentially closer links between physicians but may have underestimated the full indirect exposure of each physician, in comparison to measure of indirect exposure used in the main analysis.

Fifth, we used the share of patients with a targeted condition to measure the direct and indirect exposure of physicians in the model. This model specification tested whether spillover effects differed between physicians based on their share rather than volume of targeted patients. For example, spillovers caused by physicians reallocating their time and effort may have differed between physicians with similar volumes but different shares of targeted patients. In contrast, learning effects were more likely to have differed between physicians based on the volume of targeted patients that they treated if learning occurred from repeating incentivized tasks (i.e. learning from doing).

##### 2.4.5.3 Restricting the Estimation Sample

We also restricted the estimation sample to test further assumptions of the model. First, we excluded non‐targeted patients admitted in the quarter prior to the AQ scheme to adjust for potential anticipation effects during the pilot period of the scheme (McDonald et al. 2015).

In addition, we excluded hospital trusts in the four regions adjacent to the North‐West of England from the control group. This sensitivity test adjusted for potential between‐region spillover effects of the scheme. Between‐region spillover effects may have occurred if neighboring hospitals competed for patients based on quality (Gravelle et al. 2019) or collaborated with the incentivized hospital trusts (Baicker et al. 2013).

Finally, we estimated the analysis using a control group that excluded non‐targeted patients admitted to a hospital trust in the South‐Central and South‐East Coast regions of England. These regions implemented payment reforms in April 2010 that withheld money from hospitals failing to achieve performance targets similar to the AQ process measures. Unlike the AQ scheme, however, the same additional support was not provided to hospitals in these regions.

## 3 Results

### 3.1 Descriptive Statistics

**FIGURE 1:**
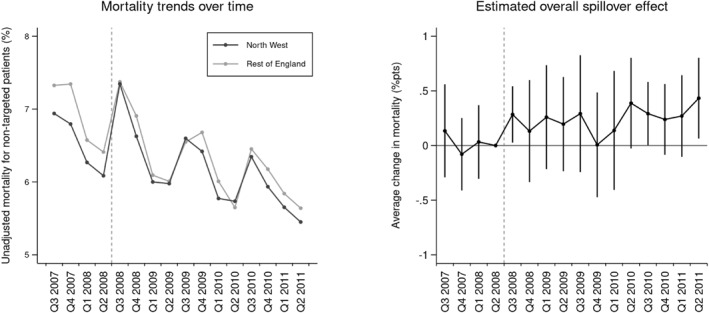
Relative changes in the mortality rate for non‐targeted patients over time.

The unadjusted mortality rate for non‐targeted patients was lower in the North‐West prior to the AQ scheme (6.5% vs. 6.9%) but declined at a slower rate over time (−0.4%pts vs. −0.6%pts) compared to the rest of the country (Table 1). This pattern is illustrated in the left panel of Figure 1, where the mortality gap between the North‐West and the rest of England is shown to narrow following the AQ scheme.

**TABLE 1:** Descriptive statistics.

	North‐west region			Rest of England		
	Before	After	Diff	Before	After	Diff
Outcome measure						
Unadjusted 30‐day mortality rate (%)	6.5	6.2	−0.4	6.9	6.3	−0.6
Physician exposure (targeted patients)						
Direct exposure	28.8	33.2	4.3	25.8	28.6	2.7
Indirect exposure	19.4	21.7	2.3	19.4	20.2	0.8
Patient level covariates						
Male (%)	45.6	45.5	−0.2	46.7	46.2	−0.5
Age ≥ 75 years (%)	36.2	36.7	0.4	38.7	38.8	0.1
Number of coexisting conditions	3.3	4.0	0.7	2.9	3.6	0.7
Income deprivation score in 2010	30.7	30.0	−0.7	23.1	23.1	−0.1
Admitted from home (%)	97.2	97.3	0.2	94.9	93.6	−1.3
Admitted via A&E (%)	71.8	74.0	2.2	66.1	68.9	2.8
Admitted at weekend (%)	11.6	12.0	0.3	11.4	11.8	0.4
Admitted in winter (%)	25.4	25.3	−0.1	24.8	25.2	0.5
Physician level covariates						
Emergency admissions per quarter	505	426	−79	268	297	29
Elective admissions per quarter	200	197	−3	184	198	14
Patients treated in contracted specialty (%)	94.0	88.7	−5.4	84.4	84.0	−0.4
Specialty level covariates						
Emergency admissions per quarter	2567	2881	313	2176	2379	203
Elective admissions per quarter	2030	2182	152	1693	1692	−1
Specialty of treatment (%)						
General medicine	44.7	48.6	3.9	46.1	43.7	−2.4
General surgery	18.3	19.8	1.5	18.9	19.4	0.5
Accident and emergency	14.4	10.5	−3.9	8.0	8.8	0.8
Geriatric medicine	8.3	6.8	−1.5	7.9	8.1	0.2
Cardiology	2.8	2.8	0.0	2.8	2.6	−0.2
Trauma and orthopedics	1.8	1.7	−0.1	2.2	2.1	−0.1
Respiratory medicine	2.4	2.4	0.0	1.8	1.9	0.0
Other	7.3	7.4	0.1	12.2	13.5	1.2
Hospital level covariates						
Emergency admissions per quarter	8186	8549	363	8005	8686	681
Elective admissions per quarter	23,286	24,441	1155	24,410	26,250	1840
Foundation trust (%)	55.0	57.1	2.2	49.2	47.8	−1.5
Trust type (%)						
Acute ‐ small	10.8	11.3	0.5	10.9	10.7	−0.2
Acute ‐ medium	36.0	34.0	−2.0	28.5	28.2	−0.3
Acute ‐ large	37.9	38.9	1.0	40.3	40.4	0.1
Teaching	15.3	15.8	0.5	20.1	20.5	0.4
Specialist	0.0	0.0	0.0	0.2	0.2	0.0
Estimation sample						
No. of admissions	149,801	149,202		781,650	769,505	
No. of physicians	915	915		5686	5686	
No. of hospital trusts	23	23		121	121	

*Note:* Estimation sample includes all admissions for a non‐targeted condition that were treated by a physician who treated at least one non‐targeted condition within the hospital in every quarter. Direct exposure measures the average number of targeted patients treated per quarter by each physician throughout the post‐policy period. Indirect exposure measures the average number of targeted patients treated per quarter by other physicians in the specialties where the physician worked.

**FIGURE A1:**
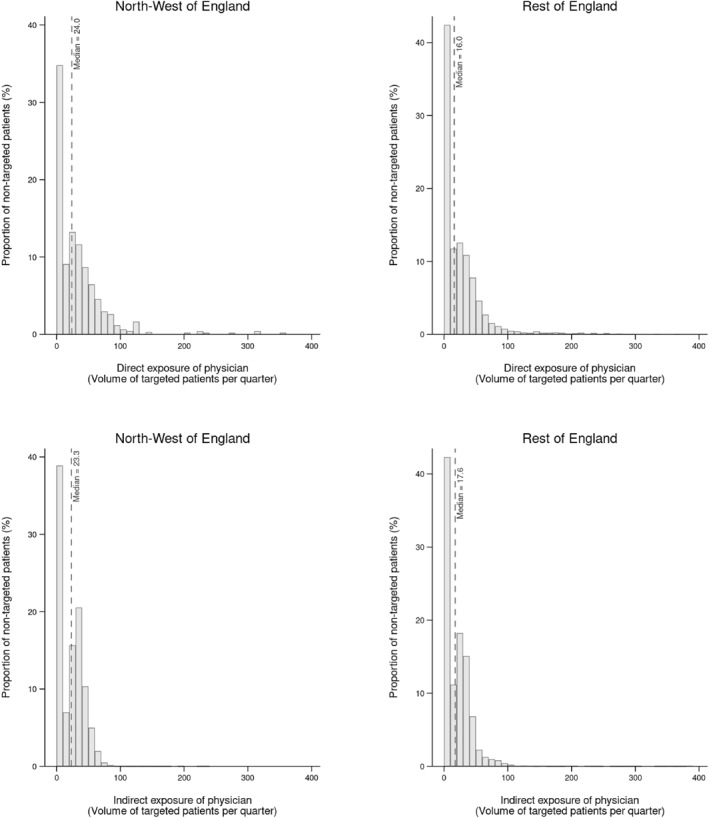
Proportion of non‐targeted patients by physician exposure to the incentives.

**FIGURE A2:**
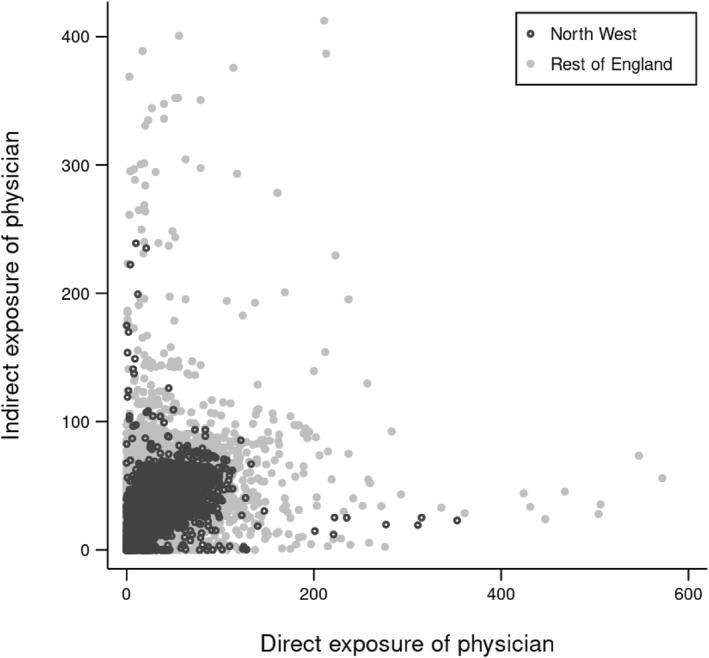
Correlation between direct and indirect physician exposure to the incentives.

The direct exposure to the incentives of physicians in the sample was higher in the North‐West than the rest of England following the AQ scheme (33.2 vs. 28.6 targeted patients per quarter) (Table 1). The indirect exposure of physicians was also higher (21.7 vs. 20.2 targeted patients per quarter). The majority of non‐targeted patients in the sample were treated by a physician with little or no direct and indirect exposure to the incentives (Figure A1). Measures of direct and indirect exposure were partially correlated in both the North‐West and rest of England (correlation coefficients = 0.36 and 0.51) (Figure A2).

Non‐targeted patients in the North‐West had higher income deprivation, more Elixhauser comorbidities, and were younger on average than in the rest of England (Table 1). They were also more likely to be admitted to hospital from their own home, during winter, and via the accident and emergency department. Hospitals in the North‐West were also smaller on average, included fewer teaching hospitals, and were more likely to be a foundation trust. Our estimation strategy adjusted for differences in these characteristics over time.

There were also differences in the characteristics of physicians and specialties. Prior to the AQ scheme, physicians in the North‐West of England were more likely to work in their contracted specialty (94.0% vs. 84.4%) and treated a higher volume of emergency patients on average (505 vs. 268 patients per quarter). Both of these measures reduced at a greater rate over time in the North‐West of England compared to elsewhere. The proportion of non‐targeted patients treated in general medicine (3.9%pts vs. −2.4%pts) and general surgery (1.5%pts vs. 0.5%pts) also increased at greater rate in the North‐West of England following the AQ scheme. These differences over time suggest evidence of a patient sorting effect.

### 3.2 Main Analysis

#### 3.2.1 Average Spillover Effect

The first column in Table 2 and the second panel in Figure 1 present estimates of the overall spillover effect from the initial DiD model. The results indicate that the average mortality rate for patients with a non‐targeted condition was 0.221% points (S.E. 0.125) higher in the North‐West following the rollout of the AQ scheme, relative to changes in mortality elsewhere. This estimated effect was equivalent to a 3.4% change relative to the mortality rate in the North‐West of England prior to the AQ scheme but was not statistically significant at the 95% confidence level. The finding suggests that the AQ scheme was associated with an adverse but statistically insignificant average spillover effect on the mortality rate for patients with a non‐targeted condition.

**TABLE 2:** Average spillover effect of the AQ scheme on the mortality rate for non‐targeted patients.

	Overall impact	Adjusting for patient sorting
Average spillover effect ($\hat{\delta }$)	0.221	0.070
	(0.125)	(0.126)
Covariates		
Patient level	Y	Y
Hospital level	Y	Y
Physician/speciality level	—	Y
Fixed effects		
Time	Y	Y
Hospital	Y	—
Physician‐hospital	—	Y
Non‐targeted patients	3,687,573	3,687,573
Pre‐trend tests	Pass	Pass

*Note:* The table presents findings from difference‐in‐differences models estimated at the patient level using ordinary least squares regression. Estimates are percentage point changes in the mortality rate for non‐targeted patients. The coefficient $\hat{\delta }$ estimates the average spillover effect of the AQ scheme on all non‐targeted patients included in the sample.

Parentheses include cluster robust standard errors at the hospital trust level. *p < 0.05, **p < 0.01, ***p < 0.001.

#### 3.2.2 Adjusting for Patient Sorting

Estimates of the average spillover effect reduced by 68% in magnitude to 0.070% points (S.E. 0.126; 1.1%) after adjusting for physician and specialty level factors and physician‐hospital fixed effects in the model (Table 2, Column 2). This change in magnitude suggests that the allocation of non‐targeted patients to different physicians and/or specialties following the introduction of the AQ scheme was associated with a reduction in mortality, explaining part of the estimated overall effect.

#### 3.2.3 Decomposing Spillover Mechanisms

The first column in Table 3 presents estimates from the full model in Equation (3). This model decomposed the average spillover effect of the AQ scheme into three components based on the exposure to the incentives of the physician.

**TABLE 3:** Decomposed spillover effect of the AQ scheme on the mortality rate for non‐targeted patients (overall and by subgroup).

	Main analysis	Subgroup analysis 1		Subgroup analysis 2	
		Non‐targeted condition in same diagnosis area	Non‐targeted condition in different diagnosis area	Hospital with above average share of targeted patients	Hospital with below average share of targeted patients
Spillover effect					
Average spillover effect with no exposure ($\hat{\theta }$)	0.321*	0.385	0.257*	0.275	0.363*
	(0.114)	(0.341)	(0.117)	(0.207)	(0.134)
Marginal spillover effect of physician direct exposure ($\hat{\mu }$)	−0.008***	−0.010	−0.006*	−0.009**	−0.006
	(0.002)	(0.005)	(0.002)	(0.002)	(0.005)
Marginal spillover effect of physician indirect exposure ($\hat{\varphi }$)	−0.001	−0.002	0.000	0.002	−0.006
	(0.004)	(0.007)	(0.004)	(0.003)	(0.007)
Patient level covariates	Y	Y	Y	Y	Y
Hospital level covariates	Y	Y	Y	Y	Y
Physician/speciality level covariates	Y	Y	Y	Y	Y
Time fixed effects	Y	Y	Y	Y	Y
Physician‐hospital fixed effects	Y	Y	Y	Y	Y
Non‐targeted patients	3,687,573	1,263,365	2,424,208	1,452,355	2,235,218
Pre‐trends tests	Pass	Pass	Pass	Pass	Pass

*Note:* The table presents findings from Equation (3), estimated at the patient level using ordinary least squares regression. Estimates are percentage point changes in the mortality rate for non‐targeted patients. The coefficient $\hat{\theta }$ estimates the average spillover effect of the AQ scheme on the mortality for non‐targeted patients who were treated by a physician with no direct and indirect exposure to the incentives. The coefficients $\hat{\mu }$ and $\hat{\varphi }$ estimate the marginal spillover effect of being treated by a physician who was directly or indirectly exposed to one additional targeted patient per quarter.

Parentheses include cluster robust standard errors at the hospital trust level. *p < 0.05, **p < 0.01, ***p < 0.001.

The estimated coefficient $\hat{\delta }$ implies that mortality was 0.321% points (S.E. 0.114; 4.9%) higher for non‐targeted patients who were treated by a physician with no direct or indirect exposure to the incentives following the AQ scheme (Table 3, Column 1). This adverse spillover effect was statistically significant and 4.6 times larger than the estimated overall spillover effect (when adjusting for patient sorting). This finding implies that resources may have been diverted away from non‐targeted patients in response to the incentive scheme.

In contrast, the estimated coefficient $\hat{\theta }$ suggests that the mortality rate for non‐targeted patients was 0.008% points (S.E. 0.002) lower for every additional targeted patient treated per quarter by their physician (i.e. as the direct exposure to the incentives of the patient's physician increased). This marginal spillover effect was equivalent to a 0.264% points (4.1%) reduction in the mortality rate for non‐targeted patients who were treated by the average physician, responsible for 33.2 targeted patients per quarter throughout the post‐policy period. This difference negated the adverse overall spillover effect observed for patients who were treated by a physician with no direct and indirect exposure to the incentives, suggesting evidence of a learning effect of the AQ scheme.

Finally, the estimated coefficient $\hat{\varphi }$ suggests that changes in the mortality rate for non‐targeted patients following the AQ scheme did not differ based on the indirect exposure to the incentives of physicians (−0.001% points; S.E. 0.004). This marginal spillover effect was small in magnitude and statistically insignificant at the 95% confidence level. This finding suggests that knowledge sharing did not occur between physicians working in the same specialty or had limited impact on the mortality rate for non‐targeted patients.

#### 3.2.4 Heterogeneous Spillover Effects

The remaining columns in Table 3 present estimates from sub‐group analyses. Estimates were larger in magnitude when estimated for patients with a non‐targeted condition in the same diagnosis area as an incentivized condition. However, the standard errors associated with these findings were also larger, partially driven by the smaller sample size. Observed spillover effects also persisted when estimated for non‐targeted patients in a different diagnosis area but were smaller in magnitude. Taken together, these findings suggest that potential spillover effects were driven by a combination of condition‐specific and general changes to care in response to the scheme.

Estimates also differed when stratified by hospitals with an above or below average share of targeted patients. The sign of the estimated average spillover effect on patients treated by a physician with no exposure remained the same in both models but was larger in magnitude and statistically significant for patients treated in hospitals with fewer targeted patients. In contrast, the estimated marginal spillover effect of a physician's direct exposure to the incentives was larger in hospitals with a higher share of targeted patients. These findings suggest that hospitals may have responded differently to the incentives depending on the proportion of their income affected by the scheme.

#### 3.2.5 Robustness Checks

##### 3.2.5.1 Pre‐Trend Tests

Trends in the mortality rate for non‐targeted patients did not differ between incentivized and non‐incentivized hospitals prior to the AQ scheme in the initial DiD model (*p*‐values = 0.62) and after adjusting for patient sorting (*p*‐value = 0.74), based on visual examinations and statistical tests. Likewise, trends in mortality rates throughout the pre‐policy period were similar for patients treated by a physician with zero direct and indirect exposure (*p*‐value = 0.45). Changes in the association between mortality and the direct (*p*‐value = 0.28) and indirect (*p*‐value = 0.55) exposure of physicians were also similar throughout the pre‐policy period. These findings were consistent across all model specifications and robust when using an alternative linear pre‐trends test.

##### 3.2.5.2 Alternative Model Specifications

Table 4 presents estimates from alternative model specifications. The estimated average spillover effect on patients treated by a physician with no exposure to the incentives increased slightly in magnitude after controlling for the exposure of the specialty in which a patient was treated (0.361% points, S.E. 0.110, 5.5%) (Table 4, Column 2). This finding suggests that increases in mortality rate for non‐targeted patients were larger in specialties with fewer targeted patients. However, the marginal benefit of being treated in a specialty with one additional targeted patient was small and statistically insignificant (−0.0002% points, S.E. 0.0002) implying that differences in the impact of the scheme between specialties were small.

**TABLE 4:** Robustness checks.

	Main analysis	Alternative model specification					Restricted sample		
		Including specialty level exposure	Including interaction b/t direct and indirect exposure	Including squared exposure terms	Using alternative measure of indirect exposure	Using share of targeted patients to measure exposure	Excluding anticipation period	Excluding neighboring regions	Excluding regions adopting similar incentives
Average spillover effect									
Physician with no exposure	0.321*	0.361*	0.284*	0.279*	0.336**	0.093	0.318*	0.294*	0.341**
	(0.114)	(0.110)	(0.112)	(0.107)	(0.115)	(0.125)	(0.112)	(0.113)	(0.121)
Marginal spillover effect									
Physician direct exposure	−0.008***	−0.008*	−0.006*	−0.021***	−0.007***	−0.023	−0.010**	−0.008**	−0.008***
	(0.002)	(0.002)	(0.003)	(0.004)	(0.001)	(0.034)	(0.002)	(0.002)	(0.002)
Physician indirect exposure	−0.001	0.000	0.000	0.016	−0.008	0.019	0.001	0.002	−0.001
	(0.004)	(0.009)	(0.009)	(0.012)	(0.009)	(0.036)	(0.003)	(0.004)	(0.004)
Specialty direct exposure		−0.0002							
		(0.0002)							
Physician direct × indirect exposure			−0.00002						
			(0.00017)						
Physician direct exposure squared				0.00008*					
				(0.00003)					
Physician indirect exposure squared				−0.00008					
				(0.00022)					
Non‐targeted patients	3,687,573	3,687,573	3,687,573	3,687,573	3,687,573	3,687,573	3,219,439	2,300,550	3,183,529
Pre‐trend tests	Pass	Pass	Pass	Pass	Pass	Pass	Pass	Pass	Pass

*Note:* The table presents alternative specifications of the main analysis. Interpretations of the average and marginal spillover effect vary depending on measures of exposure included in the model (e.g. estimates using the share of targeted patients to measure exposure denote the marginal spillover effect of being treated by a physician with a one percent point higher share of targeted patients). Parentheses include cluster robust standard errors at the hospital trust level. *p < 0.05, **p < 0.01, ***p < 0.001.

Findings from the main analysis were also consistent when testing for an interaction effect between a physician's direct and indirect exposure to the incentives (Table 4, Column 3). The estimated coefficient on the interaction term between these variables was small and statistically insignificant, strengthening the assumption that marginal spillover effects were additive and separable.

The overall interpretation of the findings also remained the same when including squared exposure terms in the model (Table 4, Column 4). In this model, however, the marginal spillover effect of being treated by a physician with higher direct exposure to the incentives was associated with diminishing reductions in the mortality rate for non‐targeted patients up to the point of treating 134 targeted patients per quarter.^3^ This finding is consistent with the hypothesis that the returns to learning were diminishing.

When using an alternative measure of indirect exposure, the estimated marginal spillover effect of being treated by a physician with higher indirect exposure was larger in magnitude but remained statistically insignificant (−0.008% points, S.E. 0.009) (Table 4, Column 5). This was expected as the alternative measure defined closer links between physicians within specialties, measuring the average exposure of physicians working in the same specialty *and* treating the same conditions. Other estimates in this model were similar to the main analysis.

Finally, estimated coefficients retained their signs but were no longer statistically significant at conventional levels when using the share of targeted patients to measure the exposure of physicians in the sample (Table 4, Column 6). This finding implies that changes in mortality differed between patients based on the volume rather than concentration of targeted patients treated by their physician. This observation was consistent with the concept of learning by doing.

##### 3.2.5.3 Restricting the Estimation Sample

Estimates were also similar after excluding patients admitted in the quarter prior to the AQ scheme to test for an anticipation effect (Table 4, Column 7). However, the estimated marginal spillover effect of being treated by a physician with higher direct exposure to the incentives was slightly larger in magnitude in this model (−0.010% points, S.E. 0.002). This finding suggests evidence of a small anticipation effect prior to the AQ scheme, potentially in response to the pilot for the AQ scheme.

Excluding hospitals in the four geographic regions surrounding the North‐West of England from the control group also had little impact on the estimates from the main analysis (Table 4, Column 8). Similarly, the sign and magnitude of the findings persisted when excluding regions that adopted similar incentives during the post‐policy period (Table 4, Column 9). These findings support the assumption that there were no between‐region spillover effects of the scheme.

## 4 Discussion

Policies in healthcare can spillover onto outcomes beyond those initially intended, affecting the overall impact of a reform. In this paper, we outlined the potential mechanisms through which spillover effects within healthcare organizations could occur and exploited detailed patient‐physician level data to estimate the spillover mechanisms of the Advancing Quality scheme; a regional quality improvement program introduced in the English NHS.

At an aggregate level, the results suggest that the AQ scheme had a small adverse spillover effect on the mortality rate for non‐targeted patients treated in an incentivized hospital. The magnitude of this estimated spillover effect offset previously observed improvements in the mortality rate for patients with a targeted condition (Kristensen et al. 2014; Sutton et al. 2012), but was statistically insignificant at the 95% confidence level.

The estimated overall spillover effect was even smaller in magnitude when adjusting for physician and specialty level factors, suggesting that part of the overall adverse spillover effect was driven by the reallocation of non‐targeted patients to different physicians and specialties. We also showed that the AQ scheme was associated with a statistically significant increase in mortality for patients treated by a physician with no direct or indirect exposure to the incentives. This adverse spillover could not have been driven by physicians substituting their effort or time. Rather, hospitals may have responded to the incentives by reallocating resources away from non‐targeted patients. This finding persisted when further adjusting for specialty exposure, suggesting evidence of an organization level response in line with qualitative evidence that hospitals adapted emergency care pathways to better identify and target incentivized patients (McDonald et al. 2015).

In contrast, patients treated by physicians with higher direct exposure to the incentives experienced a marginal reduction in mortality after the incentives were introduced. This finding suggests that physicians may have learned from the incentives, supporting qualitative evidence that physicians adapted their behavior over time (McDonald et al. 2015). Alternatively, patients treated by exposed physicians may have also benefitted from general improvements in resources allocated at the physician level. However, we suspect the former of these mechanisms to be true as the same findings were not observed when using the share of targeted patients to measure a physician's exposure, implying a volume effect consistent with concept of learning by doing more. Marginal returns to exposure were also diminishing when including a squared exposure term in the model, in line with theoretical evidence on the returns to learning (Chandra and Staiger 2007).

Finally, there was no evidence of differences in the impact of the scheme across physicians with different levels of indirect exposure to the incentives. This finding suggests that knowledge spillovers between physicians were limited, consistent with qualitative evidence which identified a lack of awareness of the incentives outside the clinical teams directly involved in the scheme (McDonald et al. 2015).

Our findings also reflect those from the wider literature, which has identified both positive and negative spillover effects of health care reforms (Francetic et al. 2022; Kondo et al. 2015). They suggest that differences in spillovers observed by previous studies may have been driven in part by differences in the spillover mechanisms affecting the analyzed population. This potential explanation has been overlooked by previous studies which have tended to evaluate spillovers as a secondary analysis without prior hypotheses about their mechanisms.

We contributed to this literature by demonstrating how clear theory driven hypotheses on the occurrence of spillover effects, combined with a credible research design, can be used to reduce the risk of wrongly attributing spurious changes in quality as spillovers. Specifically, we provided a framework for decomposing the spillover mechanisms of a reform by considering differences in the expected spillover mechanisms between physicians.

We also demonstrated how standard DiD methods could be adapted to unpick the spillover mechanisms of a reform, under additional identification assumptions. We implemented this method adjusting for a wide range of patient, physician, and hospital level covariates alongside multiple robustness tests to support the different assumptions of the model. However, we were only able to test the conditional parallel trends assumption over the course of a single year prior to the AQ scheme. As is the case with all policy analyses, we were also unable to test the parallel trends assumption during the post‐policy period as untreated potential outcomes were unobserved for hospitals in the North‐West of England following the AQ scheme. As such, we cannot rule out the possibility that other regional changes following the AQ scheme contributed to the observed effect.

Nonetheless, our study benefitted from being conducted in the context of a single payer national health system. This meant that we could be certain that policies implemented by other payers did not affect the quality of care provided to non‐targeted patients in the analysis.

Our findings highlight the importance of considering spillover effects when evaluating the impact of new healthcare reforms and serve as a warning against uncritically using patients from the same healthcare organization as a control group. We show that even using non‐targeted patients who were further removed from the targeted area of care as a control group could lead to bias estimates of the treatment effect if organization level changes were implemented in response to the reform.

The findings also have implications for the design of future healthcare reforms. First, adverse spillover effects were observed at the organizational level despite financial bonuses being paid as additional income during the first 18 months of the scheme. This finding was consistent with predictions from the theoretical literature on multitasking agency in situations where tasks are substitutes in resources, suggesting that future incentives should target specific areas of emergency care with caution. Second, the AQ scheme combined financial incentives with other support mechanisms to encourage learning, consistent with the finding that mortality was lower for non‐targeted patients treated by exposed physicians. This observation suggests that non‐financial incentives should be considered in the design of future payment reforms to maximize value for money.

## Author Contributions

All authors were involved in the conceptualization of the research. Data were curated and analyzed by P.B. P.B. and S.R.K. wrote the first draft. All authors revised and edited the manuscript and agreed on the final version. The research was supervised by Y.S.L, S.R.K., R.M., and M.S.

## Conflicts of Interest

The authors declare no conflicts of interest.

## Data Availability

The data that support the findings of this study are available from https://digital.nhs.uk/data‐and‐information/data‐tools‐and‐s. Restrictions apply to the availability of these data, which were used under license for this study. Data are available from https://digital.nhs.uk/data‐and‐information/data‐tools‐and‐s.

## Endnotes

^1^
The local organizations responsible for commissioning care in England have changed over time. Throughout our study period, care was commissioned by Primary Care Trusts (PCTs).

^2^
Patients with non‐targeted conditions as identified in previous studies were selected in the initial study by Sutton et al. (2012) to use as a control group. The conditions were selected because they had: (i) no clinical linkage to any condition included in the program, (ii) sufficient volume (more than 9000 admissions in England per year), (iii) 30‐day mortality of more than 6%, and (iv) more than 80% of deaths within 30 days after admission occurring in a hospital. The same conditions were analyzed by Kristensen et al. (2014) and Kreif et al. (2016). However, Kristensen et al. (2014) excluded patients with pulmonary embolism as these patients were targeted by a separate incentive scheme from 2010 onwards.

^3^
Note: 98.1 percent of the sample of non‐targeted patients admitted to a hospital in the North‐West of England were treated by a physician with fewer than 134 targeted patients per quarter on average throughout the post‐policy period.

## APPENDIX A

**TABLE A1:** Advancing Quality process measures for the targeted conditions that were treated on an emergency basis.

Condition	Code	Process measure
Acute myocardial infarction	NHS‐1	Aspirin at arrival
	NHS‐2	Aspirin prescribed at discharge
	NHS‐3	Angiotensin converting enzyme inhibitor (ACEI) or angiotensin receptor blocker (ARB) prescribed at discharge for patients with left ventricular systolic dysfunction (LVSD)
	NHS‐4	Adult smoking cessation advice
	NHS‐5	Beta blocker prescribed at discharge
	NHS‐6	Beta blocker at arrival (dropped from July 2009)
	NHS‐7	Fibrinolytic therapy received within 30 min of arrival
	NHS‐8	Primary percutaneous coronary intervention (PCI) received within 90 min of arrival
Heart failure	NHS‐24	Evaluation of left ventricular systolic dysfunction (LVSD) function
	NHS‐25	Angiotensin converting enzyme inhibitor (ACEI) or angiotensin receptor blocker (ARB) prescribed at discharge for patients with left ventricular systolic dysfunction (LVSD)
	NHS‐26	Discharge instructions
	NHS‐27	Adult smoking cessation advice
Pneumonia	NHS‐33	Oxygenation assessment within 24 h of arrival
	NHS‐34	Initial antibiotic selection for community‐acquired pneumonia CAP in immunocompetent patients
	NHS‐35	Blood cultures in A&E before initial antibiotic received in hospital (dropped November 2012)
	NHS‐38	Initial antibiotic received within 6 h of hospital arrival
	NHS‐39	Adult smoking cessation advice

**TABLE A2:** List of the 50 non‐targeted conditions in the sample, by diagnosis category.

Diagnosis category	Condition
Diseases of the circulatory system	Cardiac dysrhythmias
	Coronary atherosclerosis and other heart disease
	Acute cerebrovascular disease
	Other circulatory disease
	Pulmonary heart disease
	Peripheral and visceral atherosclerosis
	Aortic; peripheral; and visceral artery aneurysms
	Cardiac arrest and ventricular fibrillation
Diseases of the respiratory system	Chronic obstructive pulmonary disease and bronchiectasis
	Other upper respiratory disease
	Acute bronchitis
	Pleurisy; pneumothorax; pulmonary collapse
	Other lower respiratory disease
	Aspiration pneumonitis; food/vomitus
Neoplasms	Secondary malignancies
	Cancer of bronchus; lung
	Cancer of colon
	Leukemia
	Non‐Hodgkin's lymphoma
	Cancer of prostate
	Cancer of esophagus
	Cancer of pancreas
	Cancer of breast
	Cancer of rectum and anus
	Cancer of bladder
	Malignant neoplasm without specification of site
	Cancer of stomach
	Cancer of ovary
Metabolic diseases and immunity disorders	Fluid and electrolyte disorders
Diseases of the blood and blood‐forming organs	Deficiency and other anemia
Mental illness	Senility and organic mental disorders
Diseases of the digestive system	Gastrointestinal hemorrhage
	Biliary tract disease
	Non‐infectious gastroenteritis
	Other gastrointestinal disorders
	Intestinal obstruction without hernia
	Other liver diseases
	Liver disease; alcohol‐related
	Gastroduodenal ulcer (except hemorrhage)
	Peritonitis and intestinal abscess
Diseases of the genitourinary system	Urinary tract infections
	Acute and unspecified renal failure
	Chronic renal failure
Diseases of the skin and subcutaneous tissue	Skin and subcutaneous tissue infections
	Chronic ulcer of skin
Injury and poisoning	Complication of device; implant; or graft
	Other fractures
	Intracranial injury
Conditions and factors influencing health status	Abdominal pain
	Syncope

*Note:* Shaded cells indicate non‐targeted conditions in the same clinical area as a targeted condition.

**TABLE A3:** Representativeness of the estimation sample.

	Full sample	Estimation sample
Outcome measure		
Unadjusted 30‐day mortality rate	6.4	6.4
Physician exposure (targeted patients)		
Direct exposure	31.0	28.6
Indirect exposure	21.3	20.2
Patient level covariates		
Male (%)	46.2	46.2
Age ≥ 75 years (%)	38.8	38.4
Number of coexisting conditions	3.5	3.5
Income deprivation score in 2010	24.2	24.2
Admitted from home (%)	93.4	94.5
Admitted via A&E (%)	69.1	69.0
Admitted at weekend (%)	11.5	11.8
Admitted in winter (%)	24.8	25.1
Physician level covariates		
Emergency admissions per quarter	345	315
Elective admissions per quarter	182	195
Patients treated in contracted specialty (%)	84.9	85.1
Specialty level covariates		
Emergency admissions per quarter	2401	2404
Elective admissions per quarter	1721	1766
Specialty of treatment (%)		
General medicine	46.1	44.8
General surgery	16.7	19.3
Accident and emergency	9.6	9.1
Geriatric medicine	7.3	7.9
Cardiology	2.6	2.7
Trauma and orthopedics	2.4	2.1
Respiratory medicine	1.8	1.9
Other	13.5	12.2
Hospital level covariates		
Emergency admissions per quarter	8419	8505
Elective admissions per quarter	25,482	25,520
Foundation trust (%)	48.8	49.5
Trust type (%)		
Acute ‐ small	11.2	10.8
Acute ‐ medium	27.7	29.3
Acute ‐ large	40.5	40.1
Teaching	19.6	19.6
Specialist	0.6	0.2
Other	0.4	0.0
Sample size		
No. of admissions	5,965,840	3,687,573
% of full sample		61.8
No. of physicians	29,138	6601
No. of hospital trusts	235	144

*Note:* Full sample includes all admissions for one of the 50 non‐targeted conditions of interest used to calculate the HSMR index. Estimation sample includes all admissions for a non‐targeted condition of interest that were treated by a physician that also treated at least one non‐targeted condition within the hospital in every quarter.

Figure A1
